# *In Vivo* Induction of Oocyte Maturation and Ovulation in Zebrafish

**DOI:** 10.1371/journal.pone.0025206

**Published:** 2011-09-28

**Authors:** Toshinobu Tokumoto, Toshiya Yamaguchi, Sanae Ii, Mika Tokumoto

**Affiliations:** Department of Biology, Faculty of Science, National University Corporation Shizuoka University, Shizuoka, Japan; Texas A&M University, United States of America

## Abstract

The maturation of fish oocytes is a well-characterized system induced by progestins via non-genomic actions. In a previous study, we demonstrated that diethylstilbestrol (DES), a non-steroidal estrogen, induces fish oocyte maturation via the membrane progestin receptor (mPR). Here, we attempted to evaluate the effect of DES as an environmental endocrine disrupting chemical (EDC) upon fish oocyte maturation using live zebrafish. DES triggered oocyte maturation within several hours *in vivo* when administrated directly into the surrounding water. The natural teleost maturation-inducing hormone, 17alpha, 20beta-dihydroxy-4-pregnen-3-one (17,20beta-DHP) also induced oocyte maturation *in vivo*. Steroids such as testosterone, progesterone or 17alpha-hydroxyprogesterone were also effective *in vivo*. Further studies indicated that externally applied 17,20beta-DHP even induced ovulation. In contrast to 17,20beta -DHP, DES induced maturation but not ovulation. Theoretically this assay system provides a means to distinguish pathways involved in the induction of ovulation, which are known to be induced by genomic actions from the pathway normally involved in the induction of oocyte maturation, a typical non-genomic action-dependent pathway. In summary, we have demonstrated the effect of EDCs on fish oocyte maturation *in vivo*. To address the effects, we have explored a conceptually new approach to distinguish between the genomic and non-genomic actions induced by steroids. The assay can be applied to screens of progestin-like effects upon oocyte maturation and ovulation for small molecules of pharmacological agents or EDCs.

## Introduction

Fish oocyte maturation is triggered by the maturation-inducing steroids or substances (MIS) which induce the *de novo* synthesis of cyclin B, a regulatory component of the M-phase-promoting factor (MPF) [1], [2]. Recently, a new class of membrane-bound progestin receptor (mPR) was identified in fish and is thought to mediate the MIS signal into the oocyte [3], [4]. During the course of fish maturation, oocytes undergo drastic morphological changes associated with progression of the meiotic cell cycle, which include breakdown of the oocyte nuclear envelope (germinal vesicle breakdown, GVBD), and hydration which causes the oocyte to become transparent. Matured oocytes are then extruded from surrounding follicle cells (ovulation) to be spawned. Spawned fish oocytes instantly develop a fertilization membrane when they are released into the water regardless of whether fertilization has occurred or not.

*In vitro* assays using oocytes dissected from ovaries have shown that some endocrine disruptors induce or inhibit this biological process [5]–[7]. We have previously described the *in vitro* effects of an endocrine disrupting chemical (EDC), diethylstilbestrol (DES), upon fish oocyte maturation. Treatment of oocytes with DES alone induces maturation in both goldfish and zebrafish [6]. Furthermore, a potent inhibitory effect of PCP has been demonstrated upon oocyte maturation induced by 17,20-βDHP [7]. These results suggested that EDCs might interact with the MIS receptor. We subsequently cloned and characterized goldfish mPRα. Identification of the structure of the putative MIS receptor in goldfish as mPRα provided a means of investigating the molecular mechanism by which DES induces oocyte maturation. Binding of these EDCs to mPRα was examined using membrane fractions prepared from cultured cells transfected with the cDNA for goldfish mPRα. Specific progestin binding was measured in the plasma membranes prepared from goldfish mPRα-transfected cells [8]. Steroid competition studies showed that binding is highly specific for natural MIS, 17,20β-DHP, in membranes prepared from both ovaries and mPRα-transfected cells. DES was a relatively effective competitor of isotope-labelled 17,20β-DHP for binding to mPRα. These results demonstrate that DES can mimic the non-genomic actions of progestins by binding to mPRα. Our results clearly indicated that the membrane steroid receptor is a possible new target for endocrine disrupting chemicals (EDCs) [9].

To further investigate the effects of EDCs *in vivo*, the present study examined the effects of externally applied EDCs or steroid hormones. We showed that externally applied EDCs, as well as natural occurring steroid hormones, could induce or prevent oocyte maturation and ovulation in living fish. These findings demonstrate that environmental chemicals disrupt not only the genomic actions of steroids via nuclear receptors but also non-genomic actions via membrane steroid receptors. Furthermore, this *in vivo* assay may represent a new system for discovering genes essential for ovulation.

## Results

### Externally applied hormones and EDCs induce or inhibit oocyte maturation *in vivo*

**Figure 1:**
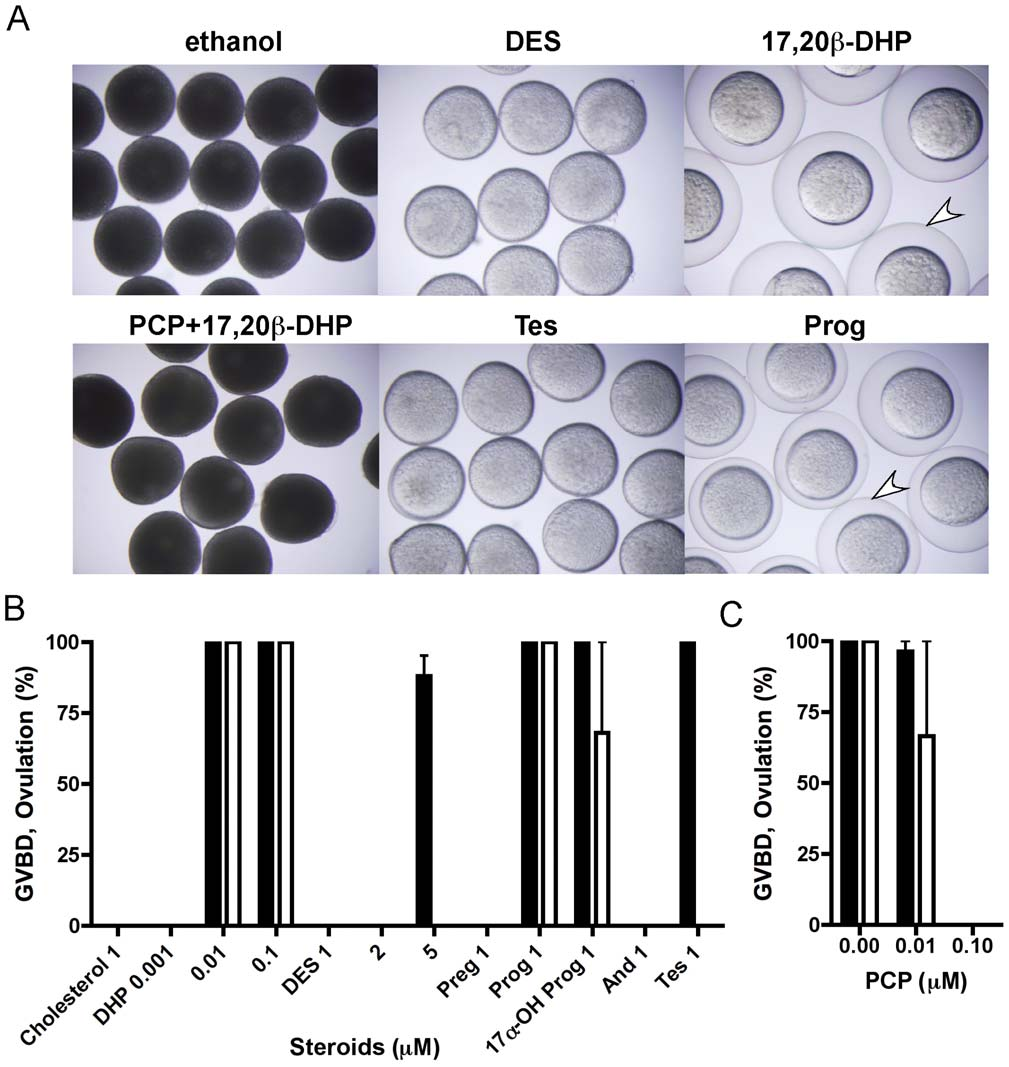
Environmental agents induce and prevent fish oocyte maturation and ovulation. Oocytes were prepared from female zebrafish after incubation with each compound for four hours. The morphology of oocytes was photographed (A); 0.01% ethanol, 5 µM DES, 0.01 µM 17,20β-DHP, 0.5 µM PCP with 0.01 µM 17,20β-DHP, 1 µM Tes and 1 µM Prog. Oocytes became transparent after treatment with 17,20β-DHP, DES, Prog or Tes but remained opaque following treatment with ethanol or PCP with 17,20β-DHP. A fertilization membrane developed in oocytes ovulated by 17,20β-DHP or Prog treatment, as indicated by the arrow. (B) Effect of various compounds on the *in vivo* induction of GVBD and ovulation. Each compound was added into the water at a final concentration of 1 mM except 17,20β-DHP (0.001, 0.01 or 0.1 µM) and DES (1, 2 or 5 µM). After four hr incubation, %GVBD (closed column) and %ovulation (open column) was determined by scoring oocytes that became transparent and formed a fertilization membrane. (C) Inhibition of *in vivo* oocyte maturation by PCP. PCP was added to the indicated concentrations, and then maturation induced by 0.01 µM of 17,20β-DHP. After further four hr incubation, %GVBD and %ovulation was assessed by scoring the oocytes. Each value represents the mean of data from three different females. Vertical lines indicate standard deviation.

To evaluate the effect of DES as an environmental endocrine disrupting chemical (EEDC) upon fish oocyte maturation, we administered DES directly into the water in which we held live zebrafish. As expected, externally applied DES induced oocyte maturation *in vivo*. We next investigated the effect of steroid hormones. Oocyte maturation was induced *in vivo* when 17,20β-DHP was added into the water, in a similar manner as when the hormone was directly administered to oocytes *in vitro*. Figure 1 shows the morphology of oocytes after four hours treatment *in vivo* with ethanol, DES, 17,20β-DHP, PCP with 17,20β-DHP, Tes or Prog. Oocytes became transparent after treatment with DES, 17,20β-DHP, Tes or Prog but remained opaque after treatment with ethanol (Figure 1A). As previously described, 17,20β-DHP- and DES-induced oocyte maturation were both prevented *in vitro* by PCP [7]. We next examined the effect of PCP on oocyte maturation *in vivo*. A combination of three different concentrations of PCP as tested against 17,20β-DHP-induced oocyte maturation *in vivo*. PCP inhibited oocyte maturation induced by 0.01 µM 17,20β-DHP (Figure 1C). This result demonstrated that PCP interacts with oocyte target molecules to inhibit oocyte maturation, as observed under *in vitro* conditions. Furthermore, oocytes from fish treated with 17,20β-DHP or Prog formed fertilization membranes immediately after transfer into the medium (Figure 1A). In most fish, ovulated oocytes (eggs) activate immediately after contact with water to form a fertilization membrane. We next estimated the percentage of ovulated oocytes by counting the oocytes that formed a fertilization membrane. Interestingly, DES and Tes induced oocyte maturation but not ovulation. As shown in Figure 1B, progestins (Prog, 17α-OH Prog and 17,20β-DHP) possessed inducing activity for both oocyte maturation and ovulation, but DES and Tes induced only oocyte maturation. Relatively higher concentrations were necessary to induce oocyte maturation *in vivo* than *in vitro*. Concentrations of 1 nM for 17,20β-DHP and 0.1 µM for DES was sufficient to induce oocyte maturation *in vitro* [6], [7] although it was necessary to increase this to 10 nM and 5 µM for *in vivo*, respectively (Figure 1B). These findings demonstrated that steroid hormones and certain kinds of EDCs penetrate the fish body, to cause effect upon oocyte maturation.

**Figure 2:**
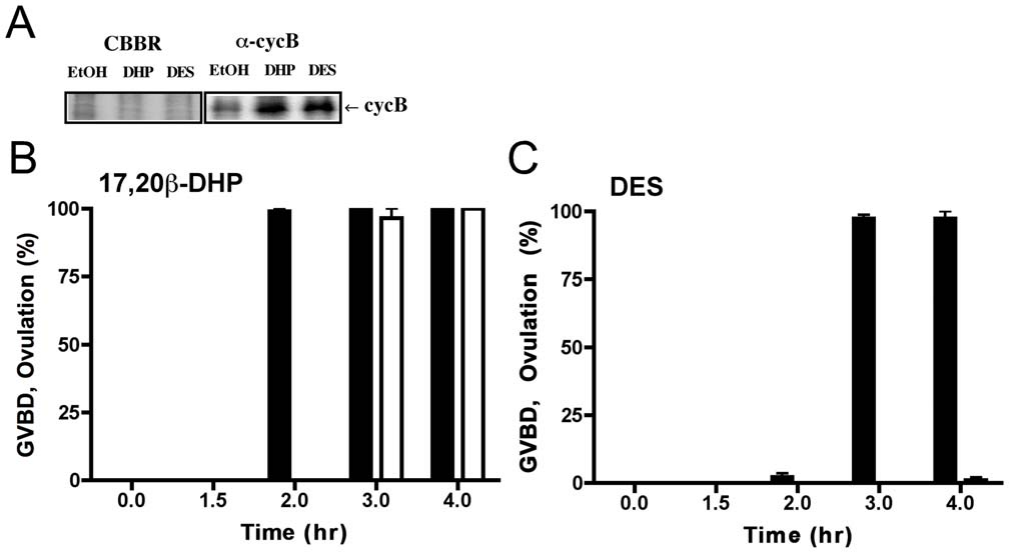
Characterization of 17,20β-DHP- and DES-induced oocyte maturation *in vivo*. (A) 17,20β-DHP and DES induce accumulation of cyclin B. Extracts were prepared from twenty oocytes after incubation with ethanol, 17,20β-DHP or DES for four hours. Extracts of each treatment were separated by electrophoresis under denaturing conditions (10.0% gel) and stained with Coomassie Brilliant Blue (CBBR), or immuno-stained with anti-goldfish cyclin B polyclonal antibody after electroblotting (α-cycB). The arrow indicates a band representing cyclin B. Time-course change of GVBD and ovulation induced by 17,20β-DHP and DES. Oocyte maturation induced by 0.01 µM 17,20β-DHP (B), 5 µM DES (C) *in vivo*. At each time point, ovarian fragments were dissected from sacrificed females and %GVBD (closed column) and %ovulation (open column) was determined by scoring oocytes that had become transparent and formed fertilization membranes. Each value represents the mean of data from three different females. Vertical lines indicate standard deviation.

**Figure 3:**
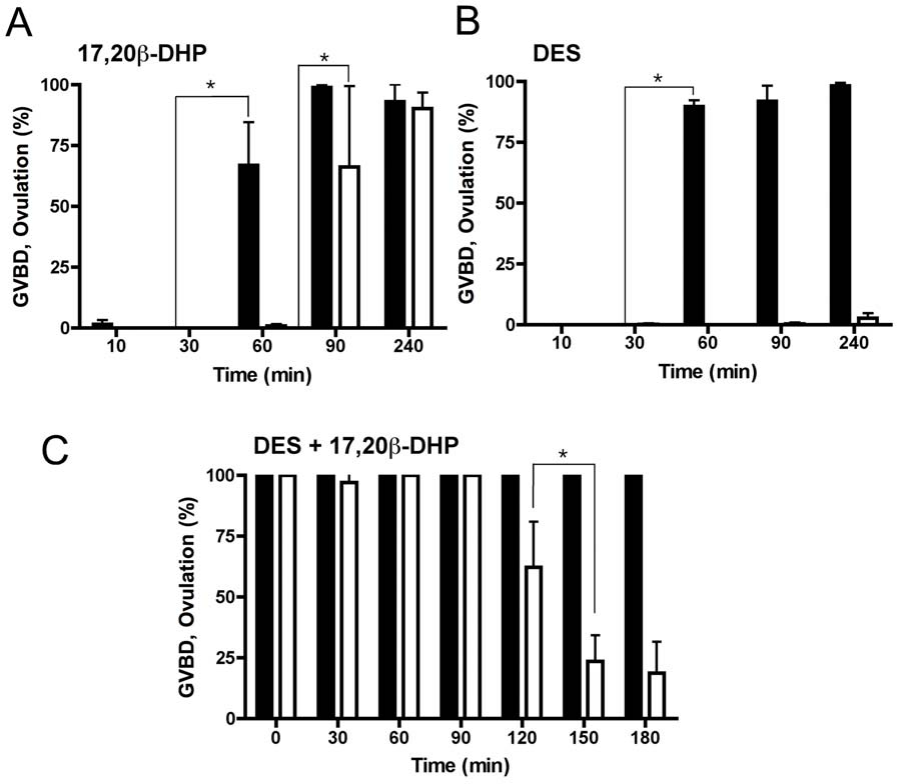
Effect of the length of treatment time of 17,20β-DHP and DES treatment during induction of oocyte maturation *in vivo*. (A, B) Each agent was added at time zero. Agents were then washed-out by changing the water three times at indicated times. After four hr incubation, %GVBD and %ovulation was assessed by scoring the oocytes. Each panel shows 17,20β-DHP (A) and DES treatment (B). (C) Effect of serial treatment with DES and 17,20β-DHP. Incubation was started by the addition of DES. Then, 17,20β-DHP was added at the indicated times. %GVBD (closed column) and %ovulation (open column) was assessed by scoring the oocytes after four hours from addition of 17,20β-DHP. Each value represents the mean of data from three different females. Vertical lines indicate standard deviation.

Immunoblot analysis clearly showed that 17,20β-DHP and DES induced accumulation of cyclin B protein (Figure 2A), a well-characterized intracellular molecular event that results in an elevation of MPF kinase activity. The time course of oocyte maturation induced by 17,20β-DHP and DES *in vivo* (Figure 2B and C) was the same as that induced by both reagents *in vitro* [6]. Although 17,20β-DHP induced ovulation too, this occurred one hour after the completion of oocyte maturation. The minimum duration of drug-treatment required to induce oocyte maturation was 60 min for 17,20β-DHP- and DES-treatment (Figure 3A and B). To induce ovulation, more than 90 min was necessary. These results suggested that both compounds penetrate the fish body at the same rate but oocyte maturation and ovulation is induced by distinct pathways and relatively longer periods of time are necessary to activate the ovulation-inducing pathway than the pathway necessary for oocyte maturation. It was demonstrated that DES does not possess the inhibitory effect on ovulation by the treatment with mixture of DES and 17,20β-DHP (Figure 3C). In order to induce ovulation, it was necessary to add 17,20β-DHP within 120 minutes of DES-treatment (Figure 3C). Addition of 17,20β-DHP after 150 min did not lead to the induction of ovulation. This indicated that oocyte maturation and ovulation should occur in a serial manner and coinside induction is necessary for ovulation with oocyte maturation.

### 17,20β-DHP induces natural ovulation *in vivo*

**Figure 4:**
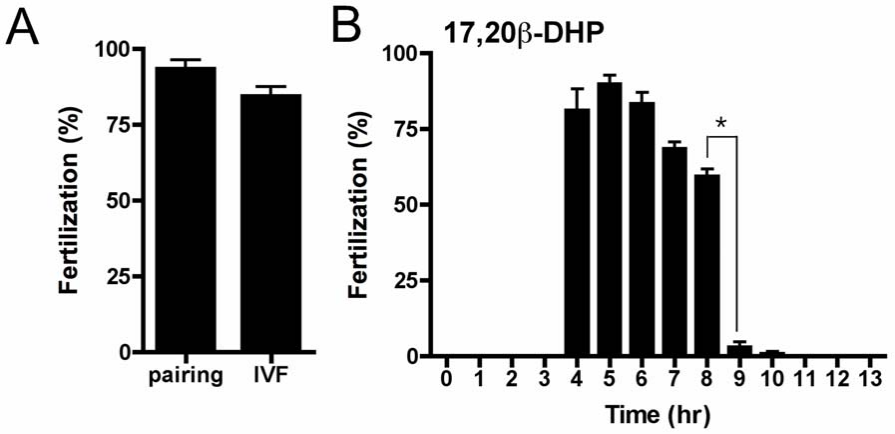
Externally applied 17,20β-DHP induced natural spawning. (A) Fertilization rates were compared between eggs squeezed from females treated with *in vivo* 17,20β-DHP and eggs resulting from normal pairing. Three spawning pairs of zebrafish were selected by standard pairing techniques and checked for fertilization rate. Several days after spawning, when ready for their next spawning event, females were treated with 17,20β-DHP and artificial fertilization conducted upon squeezed eggs using sperm obtained from the paired males. (B) Treatment of more than thirty female zebrafish with 17,20β-DHP *in vivo* began at time zero. During incubation, three females were selected and artificial fertilization conducted for eggs squeezed at each time point. Fertilization rates were assessed by counting the proportion of eggs dividing into the 4-cell stage after fertilization. Each value represents the mean of data from three different females. Vertical lines indicate standard deviation.

To demonstrate that ovulated oocytes arising from 17,20β-DHP treatment were normal or not, we conducted *in vitro* fertilization. The spawning cycle of zebrafish is reported as 2 to 5 days intervals depending on the temperature [10], [11]. And surge of hormones to induce oocyte maturation and ovulation begin during the night on the day before spawning to prepare the eggs to be spawn after the onset of lights [12]. We selected gravid female without ovulated eggs in the morning 1 to 2 hrs later of onset of lights. Thus the female used in this study should be out of natural spawning cycle and oocyte maturation and ovulation were induced artificially by the compounds added into the water. Eggs ovulated following treatment with 17,20β-DHP were indeed fertilizable (Figure 4). When artificial fertilization was conducted each hour after administration of 17,20β-DHP, we found that fertilizable oocytes were obtained until 8 hours after the addition of 17,20β-DHP. This indicated that zebrafish could retain fertilizable oocytes for approximately 5 hours after ovulation. *In vitro* fertilized eggs developed normally and were fertile (Data not shown). These results showed that 17,20β-DHP-induced ovulation was identical to physiological ovulation and that this procedure can be used as a new technique for artificially inducing ovulation in fish.

## Discussion

We previously described the induction of oocyte maturation in fish by an endocrine-disrupting chemical, diethylstilbestrol (DES) [6], a non-steroidal estrogen, as well as prevention by pentachlorophenol (PCP) [7], a widely used biocide. We demonstrated that the agonistic effect of DES occurs via the mPR by demonstrating a direct interaction between DES and mPR with steroid binding assays using recombinant protein expressed in culture cells [8]. We next tried to address whether DES acts upon oocyte maturation through the surrounding aquatic environment by simply adding a solution of DES into the water in this study. Results showed that fish oocytes kept in water containing DES successfully underwent induced oocyte maturation and became transparent, a morphological feature typical of matured oocytes. Oocyte maturation was induced *in vivo* by this simple method within several hours (2 hours by 17,20β-DHP and 3 hours by DES), as *in vitro* studies. Since the time taken to induce oocyte maturation was not significantly different between *in vitro* and *in vivo* treatments, it can be concluded that steroids and EDCs penetrate the fish body very rapidly and act directly on the ovaries (oocytes and follicle cells). PCP, which showed a potent inhibitory effect *in vitro*, also prevented oocyte maturation *in vivo*. Thus, it is suggested that environmental EDCs disrupt the spawning of fish by affecting the pathway for non-genomic actions of steroids. Whilst a number of reports have described the disruption of genomic actions of steroids by EDCs (for review see [9]), only a very small proportion has investigated the disruption of non-genomic actions. Reports describe antagonistic and agonistic pathways involved in MIS-induced oocyte maturation [5]–[7], [13], [14]. Our findings in this study demonstrate that environmental chemicals disrupt not only the genomic actions of steroids via nuclear receptors but also non-genomic actions via membrane steroid receptors. The new simple method established in this study may be applicable to evaluate the effect of environmental endocrine disrupting chemicals (EEDCs) upon non-genomic actions of progestins by the assessment of fish oocyte maturation.

We also investigated the effect of natural MIS, 17,20β-DHP, upon *in vivo* oocyte maturation in fresh water fish. 17,20β-DHP also induced oocyte maturation *in vivo*. Furthermore, we found that prolonged incubation with 17,20β-DHP induced ovulation *in vivo*. Matured oocytes can be squeezed from zebrafish which have been treated with 17,20β-DHP for more than 3 hours. Squeezed oocytes successfully developed a fertilization membrane immediately after contact with water. This is a natural characteristic of ovulated fish oocytes which are automatically activated by water contact and begin development without insemination. Although DES induced oocyte maturation, and oocytes became transparent, we found that this chemical could not induce ovulation, even after long periods of incubation. Although oocyte maturation and ovulation are physiological processes that occur in a serial manner in order to produce fertilizable eggs, it is apparent that oocyte maturation is induced by non-genomic steroid actions whilst ovulation is induced by genomic actions. Immature oocytes within the ovary underwent induced oocyte maturation in response to 17,20β-DHP [15], [16]. 17,20β-DHP is secreted from follicular cells and acts on mPRs upon the plasma membrane of oocytes inducing maturation via non-genomic actions. Matured oocytes ovulate by the disruption of follicle cells surrounding the oocytes. In contrast to oocyte maturation, the ovulation-inducing pathway is thought to be activated by genomic actions because Actynomycin D inhibits the production of mRNA, thus preventing ovulation in fish [16]. Following ovulation, oocytes are spawned and fertilized for development. Although metalloproteinases responsible for the rupture of follicle cells were identified in Medaka [17] and gene lists of up- or down-regulated during fish ovulation were reported [18], [19], the molecular mechanism of ovulation is still largely unknown beside its importance in design of drugs for complications of pregnancy. In this study we discovered a novel method to induce *in vivo* maturation without ovulation using DES or Tes. We also discovered a way to induce ovulation using 17,20β-DHP in the same manner. This allowed us, for the first time, to collate a list of genes which are specifically up- or down-regulated in order to induce ovulation by comparing the expression levels of genes between 17,20β-DHP-treated sample and DES- or Tes-treated ones. We are now trying to identify genes for the ovulation-inducing cascade. An efficient *in vivo* assay will be highly useful in identifying key regulators for ovulation. Furthermore the method also applicable to evaluate the effect of EEDCs upon genomic actions of progestins by the assessment of ovulation.

## Methods

### Ethics Statement

All animal experiments were conducted according to relevant national and international guidelines ‘Act on Welfare and Management of Animals’ (Ministry of Environment of Japan). Ethics approval from the local IACUC was not sought since this law does not mandate protection of fish.

### Materials

Zebrafish were raised and kept under standard laboratory conditions. Fish used for experiments were maintained in an out-flow culture system maintained at 28.5°C on a 14 h light/10 h dark cycle [20]. Steroid hormones (Progesterone, testosterone, 17α-hydroxyprogesterone, 17, 20β-DHP) and DES were purchased from Sigma Chemical Co. (St. Louis, MO). PCP was obtained from Wako Pure Chemical Industries (Osaka, Japan).

### *In vivo* assay of oocyte maturation and ovulation

Gravid female zebrafish possessing full-grown immature oocytes were selected from a mixed group of 10–50 males and females held in 20 cm×25 cm square acryl case which was 25 cm high and fed with continuous out-flow water. Females were transferred into a glass case containing 100 ml of water per fish. Fish were exposed to agents *in vivo* by adding each agent into the water (from a 10,000-fold stock in ethanol) at 28.5°C. After incubation, zebrafish ovaries were isolated from sacrificed females and placed in fresh zebrafish Ringer's solution (116 mM NaCl, 2.9 mM KCl, 1.8 mM CaCl_2_, and 5 mM HEPES, pH 7.2). Ovaries were dissected manually into ovarian fragments (each containing 1–10 oocytes) using fine forceps. Oocyte morphology was photographed under a binocular microscope (SZX12, Olympus, Japan). GVBD was assessed by scoring the oocytes that became transparent [21]. Ovulation was assessed by scoring oocytes that showed a clear fertilization membrane. The rate of GVBD and ovulation were determined in at least twenty oocytes.

### Western blot analysis

Intact follicles were carefully isolated using fine forceps. Groups of twenty intact follicles were transferred to a 1.5-ml Eppendorf micro centrifuge tube and crushed with 5 strokes of a plastic pestle in 200 µl of SDS-PAGE sample buffer. Samples were centrifuged at 5,000 rpm for 5 min at 4°C in a fixed-angle rotor (MX-300 microcentrifuge, TOMY, Tokyo, Japan). The supernatant (100 µl) was collected for electrophoresis and immunoblotting. Ten microliter samples were resolved in 10% SDS-PAGE gels and transferred to a nitrocellulose membrane for Western blot analysis. Membranes were incubated overnight with blocking solution (5% non-fat milk in TBST buffer: 50 mM Tris/100 mM NaCl/0.1% Tween 20, pH 7.4). Membranes were then washed with TBST buffer and incubated for 1 hr at room temperature with cyclin B antibodies diluted 1,000-fold in TBS buffer (50 mM Tris/100 mM NaCl, pH 7.4). Membranes were then washed with TBST buffer, and incubated for 1 hr at room temperature with horseradish peroxidase conjugated to goat anti-guinea pig antibody (Zymed). Blots were washed three times for 5 mins with TBST buffer at the end of the incubation period, treated with enhanced chemiluminescence (Pierce), and finally exposed to X-ray film.

### Fertilization

The fertilization ability of ovulated oocytes was assessed by *in vitro* insemination following standard methods [20]. The fertilization rate (%) was calculated by determining the percentage of embryos developing to 4-cell or subsequent stages. The rate of fertilization was determined in at least twenty embryos.

### Statistical Analysis

All experiments were repeated three times. One-way analysis of variance (ANOVA) was calculated analyzed using GraphPad Prism (San Diego, CA). A *P* value<0.05 was considered statistically significant.
